# Incremental Validity of ADHD Dimensions in the Predictions of Emotional Symptoms, Conduct Problems, and Peer Problems in Adolescents Based on Parent, Teacher, and Self-Ratings

**DOI:** 10.3390/pediatric16040095

**Published:** 2024-12-10

**Authors:** Rapson Gomez, Taylor Brown

**Affiliations:** 1 School of Health Sciences and Psychology, Federation University, Ballarat, VIC 3350, Australia; 2 Applied Health, School of Health and Biomedical Science, RMIT University, Melbourne, VIC 3001, Australia

**Keywords:** ADHD dimensions, emotion symptoms, conduct problems, peer problem, incremental validity

## Abstract

**Background:** The present study investigated the incremental validity of the ADHD dimensions of inattention (IA), hyperactivity (HY), and impulsivity (IM) in the predictions of emotion symptoms (ESs), conduct problems (CPs), and peer problems (PPs) in adolescents based on parent, teacher, and self- ratings. **Method:** A total of 214 ratings were collected from adolescents, their parents, and teachers in Australia. A structural equation modeling approach was employed to evaluated incremental validity. **Results:** The findings revealed that, controlling for gender, IM contributed moderate, low, and low levels of variance in predicting ESs based on parent, teacher, and self-ratings, respectively. Additionally, IM contributed moderate, substantial, and moderate levels of variance to CP predictions based on parent, teacher, and self-ratings, respectively. Furthermore, after controlling for gender, IM, and HY, parent-rated IA contributed a low level of variance to the prediction of ESs, while teacher and self-rated IA did not contribute significantly to the prediction of ESs, CPs, or PPs. **Conclusions:** The findings underscore the differential predictive validity of ADHD dimensions across informants and outcomes, highlighting impulsivity’s stronger association with conduct problems and emotional symptoms. These results have theoretical and practical implications for understanding ADHD-related risks in adolescence and tailoring interventions accordingly.

## 1. Background

Attention-Deficit Hyperactivity Disorder (ADHD), as defined by the American Psychiatric Association [1], is a prevalent and complex neurodevelopmental disorder affecting many children and adolescents worldwide [2]. According to the Diagnostic and Statistical Manual of Mental Disorders, Fifth Edition, Text Revision (DSM-5-TR; [1]), ADHD is characterized by 18 symptoms, which remain consistent across DSM-5 [3], DSM-IV-TR [4], and DSM-IV [5]. These symptoms span three dimensions, including inattention (IA; nine symptoms), hyperactivity (HY; five symptoms), and impulsivity (IM; three symptoms), with HY and IM symptoms often considered together for diagnostic purposes as hyperactivity/impulsivity (HI). Notwithstanding this, existing findings and theory are supportive for separate IA, HY, and IM factors [6]. To date, there has been only limited exploration of how these different ADHD dimensions are associated with the dimensions reflecting disorders with which ADHD is highly comorbid, such as conduct and emotional disorders. The goal of the current study was to examine, in a group of adolescents, the incremental validity of the ADHD dimensions in the prediction of conduct, emotional, and peer problems.

## 2. Relationships Between the ADHD Dimensions and Relevant Psychopathology Dimensions

ADHD is highly comorbid with both externalizing disorders and internalizing disorders [7,8,9]. According to Gnanavel et al. [10], approximately 60% to 100% of children with ADHD are diagnosed with one or more comorbid conditions, including autism spectrum disorder, learning disorders, tic disorders, depressive disorder, bipolar disorder, anxiety disorders, conduct disorder, and oppositional defiant disorder [10,11,12]. Given this, it is plausible that each ADHD dimension (i.e., IA, HY, and IM) is uniquely associated with specific comorbid conditions.

Research suggests that internalizing behaviors, such as anxiety [13,14,15] and depression [16], are more strongly linked to IA symptoms than HY or IM symptoms. In contrast, externalizing behavior, including aggression, are more closely associated with HI symptoms, which combines HY and IM (e.g., [17,18,19,20]). Additionally, ADHD, particularly the HI symptoms, has been linked to interpersonal behavior problems [21,22,23]. While most studies to date have examined IA and HI as broad categories, fewer studies have explored the distinct contributions of IA, HY, and IM individually to these comorbid disorders [24,25,26].

McKee [25] investigated the relationship between IA, HY, and IM dimensions and various psychological outcomes, including anxiety, depression, disruptive behavior, and peer relationships. After accounting for the shared variance among these dimensions, the study found that HY and IA were specifically associated with anxiety, IA and IM were linked to disruptive behavior, and IM was uniquely associated with poor peer relationships. Similarly, Muris and Meesters [26] explored how the ADHD dimensions relate to the dimensions in the Child Behavior Checklist (CBCL) and the Youth Self-Report (YSR; [27,28]). The findings showed that IM had stronger associations with aggressive behavior and delinquent behavior compared to IA and HY. Although all three dimensions were connected to YSR aggressive behavior, the associations for HY and IM were notably stronger than those for IA. Particularly, after controlling for the shared variance among the ADHD dimensions, HY and IM remained significantly associated with aggressive behavior, whereas the association between IA and aggressive behavior was close to zero. Furthermore, IM was more closely linked to YSR delinquent behavior than IA and HY. While IA and HY were more strongly related to attention problems, IM had a strong connection to aggressive and delinquent behaviors.

The study by Gomez et al. [24] examined the association between ADHD symptom groups and various psychological outcomes, including hyperactivity, emotional symptoms, conduct problems, peer problems, and prosocial behavior, using the Strength and Difficulties Questionnaire (SDQ; [29]). The study employed a CFA S-1 model [30] to capture the relationship more accurately between ADHD dimensions and these outcomes. In this model, one symptom group is selected (typically based on theoretical model) as the reference indicators for the general factor. The chosen group of reference items loads only on the general factor, without having their own specific factor. For example, if IM is selected as the reference, the variance in the general factor reflects the variance of the IM group. Other symptom groups are modeled as specific factors that correlate with the general factor but retain a unique residual variance, which is interpreted as the variance not shared with the general factor.

Aligning with trait impulsivity theory [31] and other studies in this area [32,33], Gomez et al. [24] used the IM symptoms to model the general factor. Thus, the general factor’s variance was considered equivalent to that of the IM factor. The study’s findings demonstrated that for parent ratings, the general factor (IM factor) was a significantly positive predictor of all the SDQ factors, i.e., emotional symptoms, hyperactivity, conduct problems, peer problems, and prosocial factors. In addition, the IA-specific factor was positively associated with hyperactivity, peer problems, and prosocial, while the HY-specific factor did not predict any of the SDQ factors. For teacher ratings, the general factor (IM factor) was, again, positively associated with hyperactivity, conduct problems, peer problems, and prosocial factors but not emotional symptoms. As with parent ratings, the IA-specific factor predicted hyperactivity and prosocial factors, whereas the HY-specific factor was not a significant predictor of any SDQ factors.

While previous studies have provided valuable insights into how specific ADHD dimensions are uniquely associated with psychological outcomes, they do not examine the incremental validity. Unique associations reveal the relationship between the predictor and the outcome in isolation, whereas incremental validity refers to the additional unique variance a predictor contributes to the outcome after accounting for other relevant variables [34]. This type of analysis helps determine the relative importance of each predictor among a set of variables in explaining the outcome. In other words, incremental validity reflects the “bonus” predictive power gained by adding a new predictor to the model [35]. The value of incremental validity lies in its ability to clarify critical relationships between predictors and outcomes, thereby facilitating the design of more targeted interventions [36]. For example, Pritchard et al. [36] demonstrated how incorporating neuropsychological assessments provides additional insights when evaluating youth with ADHD, demonstrating the practical utility of incremental validity in clinical settings.

In the context of ADHD dimensions, incremental validity helps answer questions, such as “does IA predict an outcome or criterion (for example, conduct problems), controlling for IM and HY?”. Findings from such analyses may offer important clinical insights [34,37]. For instance, if IM is found to predict conduct problems after controlling for HY and IA, clinicians might prioritize IM-focused assessments and interventions to better manage potential comorbidities. Conversely, if HY proves to be a significant predictor after controlling for IM and IA, clinical focus should shift towards HY-targeted strategies to address underlying conduct issues.

## 3. Modeling the Incremental Validity of the ADHD Dimensions in Predicting Outcomes

Incremental validity refers to the additional variance a variable contributes to an outcome, beyond what is explained by other relevant variables [38]. For ADHD dimensions, the incremental validity of each dimension depends on which other ADHD dimensions are controlled [39]. For example, the incremental validity of IM will differ when only IA is controlled compared to when both IA and HY are controlled. While it is statistically possible to explore the incremental validity of any variable under multiple control conditions, such an extensive exploration can lead to complexity and difficulty in interpreting findings [38]. Therefore, the focus should be on theoretically and clinically meaningful evaluations that are best achieved by progressively controlling for the more central ADHD dimensions and prioritizing their relative importance.

In Barkley’s [40] inhibitory control model, IM is considered as a superordinate factor that underlies several executive functions (e.g., working memory, planning, regulation of arousal, emotion, and motivation). According to this model, ADHD arises from deficiencies in executive functions, with poor inhibitory control at its core. In contrast, the dual-pathway model of ADHD, proposed by Sonuga-Barke [41], emphasizes two independent deficits, motivation and executive (related to response inhibition) functions. This model highlights distinct pathways: motivation deficits are linked to HI, while executive function deficits are connected to IA [42]. Unlike single-pathway models, which do not distinguish between IA and HI, the dual-pathway model acknowledges these two dimensions as separate processes contributing to ADHD.

The trait impulsivity theory [31,43] suggests that a highly heritable latent trait of impulsivity increases one’s vulnerability to externalizing disorders, including ADHD. At extreme levels, this trait manifests as impulsivity, characterized by a preference for immediate rewards over larger delayed rewards, impulsive actions, poor planning, and deficits in self-control [31]. From a development perspective, this impulsivity trait first contributes to HI symptoms during preschool years [31,44]. IA symptoms typically emerge later, around school entry, and are secondary to HI symptoms [45].

By integrating the trait impulsivity theory with Barkley’s inhibitory control model and the dual pathway of the ADHD model, it can be inferred that while both the IA and HI symptoms are core ADHD symptoms, HI symptoms may represent the primary underlying symptom group. Notably, the trait impulsivity theory does not distinguish between HY and IM symptom groups. However, research using confirmatory factor analysis (CFA) provided strong support for a three-factor (IA, HY, and IM) model over a two-factor (IA and HI) model [46,47,48], suggesting that HY and IM should be treated as distinct factors. Further evidence indicates that HY primarily results from difficulties inhibiting motor responses or managing impulsivity [49,50], implying that HY develops secondary to IM. In other words, IM holds greater importance than HY. Given that IA symptoms are thought to emerge secondary to both IM and HY, it can be reasoned that IM is the critical symptom group, followed by HY and then IA.

Thus, IM is likely the most important predictor of ADHD-related outcomes, with HY and IA making additional but secondary contributions. Specifically, HY contributes beyond IM, and IA adds variance beyond HY. For a comprehensive theoretical and clinical evaluation of ADHD, it is essential to examine the incremental contribution of HI symptoms while controlling for IM, as well as the contribution (incremental contribution) of IA while controlling for HY.

## 4. Methodology for Examining Incremental Validity

Incremental validity is traditionally assessed using hierarchical regression analyses. In this approach, the predictors are entered in a prespecified order across multiple steps based on the study’s aim. After each step, the changes in *R*^2^ are calculated, and the corresponding *F*-statistic is tested for significance. A significant *F* indicates that the predictor (or group of predictors) in that step contributes an additional unique variance beyond what was explained by the predictors entered in earlier steps.

While hierarchical regression is straightforward, easy to implement, and easily interpreted, it has several limitations. According to Feng and Hancock [51], it is a multistep process that can be cumbersome. Additionally, since both predictors and outcomes are treated as observed variables, they may contain random errors, compromising the independence of the constructs and introducing potential bias. Another drawback is that hierarchical regression is inflexible, as it cannot simultaneously analyze multiple outcomes.

To overcome these limitations, Feng and Hancock [51] introduced a structural equation modeling (SEM) approach for evaluating incremental validity. This method offers greater flexibility by allowing multiple outcomes to be examined concurrently and reduces potential bias. Given these advantages, it is believed that SEM is the superior alternative to traditional hierarchical regression for evaluating incremental validity. Further details about the SEM approach are provided in the Section 6.4.

## 5. Aims of the Study

Building on the existing literature, the major aim of this study was to apply the SEM method developed by Feng and Hancock’s [51] to determine whether HY offered incremental variance over IM in predicting key outcomes. Additionally, the study sought to assess whether IA provides incremental variance over HY and IM for the same outcome variables. Incremental validity was evaluated separately using parent, teacher, and self-ratings of Australian adolescents. The outcome dimensions (i.e., emotional symptoms, conduct problems, and peer problems) were measured through the corresponding subscale in the SDQ [29] for each informant group. Since gender is known to influence ADHD symptoms presentations (e.g., [52]), it was included as a covariate in all the analyses, preceding the ADHD dimensions.

As in the study by Gomez et al. [24], which explored ADHD dimensions that correlate with the SDQ scales, these are particularly relevant in the current study. Although the study did not address incremental validity, its results, combined with the theoretical framework discussed, informed the current hypothesis regarding the relative importance of the ADHD dimensions. Specifically, following the trait impulsivity model and the findings of Gomez et al. [24], it was expected that across parent-, teacher-, and self-rated IM, all the outcome variables (i.e., emotional symptoms, conduct problems, and peer problems) would be predicted, controlling for gender; HY would not contribute additional variance to any of the outcome variables beyond IM and gender; and IA would contribute additional variance to emotional symptoms, but not to conduct problems and peer problems, after controlling for gender, IM, and HY.

## 6. Method

### 6.1. Participants

The sample comprised 214 adolescents (females = 111; males = 104) whose ages ranged between 12 and 17 years. The mean age was 13.82 years (*SD* = 1.30 years), with no significant differences between males (M = 13.90, *SD* = 1.35) and females (M = 13.74, *SD* = 1.35), with *t* (212) = 0.88, *ns*. The participants were drawn from diverse geographical locations, with 43.9% from metropolitan areas, 18.2% from regional areas, and 37.9% from rural regions in Australia. Their socioeconomic status was assessed through the occupational classification of parents, primarily based on the father’s occupation (or mother, when the father was not available).

Occupations were coded according to the Australian Standard Classification of Occupations (ASCO; [53]), which includes nine major hierarchical categories defined in terms of occupational levels related to skills and specialization. In decreasing order, they are managers and administrators (coded 1); professionals (coded 2); associate professionals (coded 3); tradespersons (coded 4); advanced clerical and service workers (coded 5); intermediate clerical, sales, and service workers (coded 6); intermediate production and transport workers (coded 7); elementary clerical, sales, and service workers (coded 8); and laborers (coded 9). Those not employed were also coded 9 in this study. The overall mean occupational level of fathers for 187 participants, for whom data were available, was 4.80 (*SD* = 3.08), representing the middle social class on the ASCO scale. The ethnic composition of the sample was predominantly European (more than 94%), with additional representation from Indigenous, Asian, Middle Eastern, and African backgrounds.

All the adolescents in this study had also participated in one or more prior studies that examined various aspects of ADHD, including the factor structure and gender invariance of the adolescent self-rating version of the DBRS [54]; the agreement levels between adolescent self-ratings and mother or teaching ratings, which were analyzed using the correlated trait-correlated method minus one model [55]; and the item response theory (IRT) properties of ADHD symptoms, which were evaluated using the graded response model [56]. Therefore, the objectives in these past studies were completely different from those of the current study.

### 6.2. Measures

Then mothers completed a demographic questionnaire that collected information on their age, gender, ethnicity, country of birth education, and employment. Mother and teacher ratings of their adolescents’ ADHD symptoms were obtained using the appropriate version of the Disruptive Behavior Rating Scale (DBRS; [57]). The adolescents also provided self-ratings for their ADHD symptoms using a version of the DBRS [55] adapted for self-reports. Additionally, the mothers, teachers, and adolescents completed the respective parent and adolescent versions of the Strength and Difficulties Questionnaire (SDQ; [29]).

#### 6.2.1. DBRS [57]

The DBRS was used to assess the core ADHD symptoms of IA and HY/IM. Both the parent and teacher versions included the nine DSM-IV IA symptoms and nine DSM-IV HY/IM symptoms, with the word “often” omitted from each symptom description. The respondents were asked to circle the number that best describes the child’s behavior over the past six months. Ratings were provided on a four-point scale (0 = never or rarely, 1 = sometimes, 2 = often, and 3 = very often). Cronbach’s alphas for the mothers’ ratings of the IA, HY, and IM scales were 0.92, 0.85, and 0.88, respectively, while the teachers’ ratings were 0.96, 0.90, and 0.86, respectively.

In the adolescent self-report version, the instructions were adjusted to make a scale more suitable for self-assessment. For example, the general instructions asked adolescents to “Circle the number that best describes your behavior over the past six months”. Additionally, item wording was modified to reflect self-referential behavior. For instance, the first IA symptom “fails to give close attention to detail or makes careless mistakes in schoolwork” in the parent version was changed to “fails to give close attention to detail or makes careless mistakes in my schoolwork” in the adolescent self-rating version. Like the parent and teacher versions, the adolescents rated the presence of each symptom on a four-point scale (0 = never or rarely, 1 = sometimes, 2 = often, and 3 = very often). For adolescent self-ratings, the Cronbach’s alphas for the IA, HY, and IM scales were 0.84, 0.73, and 0.72, respectively.

#### 6.2.2. SDQ [29]

The SDQ was administered in its self-rating (adolescent), parent, and teacher rating versions [29]. All three versions contain 25 items across five scales, including hyperactivity, emotional symptoms (ESs), conduct problems (CPs), peer problems (PPs), and prosocial behavior. Each scale consists of five items, and respondents rated each item as either “not true” (scored 0), “somewhat true” (scored 1) or “certainly true” (scored 2). For the purpose of the present study, the ES, CP, PP scales were used to model these respective outcomes in the incremental validity analysis. The internal consistency (Cronbach’s alpha) values for the mothers’ ratings for the ES, CP, and PP scales in this study were 0.74, 0.69, and 0.72, respectively; for the adolescents’ self-ratings, 0.79, 0.71, and 0.65, respectively; and for the teachers’ ratings, they were 0.66, 0.66, and 0.57, respectively.

### 6.3. Procedure

The study received approval from the Human Ethics Research Committee of the University of Ballarat (Australia), adhering to the ethical standards and guidelines established by the (Australian) National Medical Research Council. A stratified random sampling method was employed to select the schools for participation, creating nine groups, each presenting a region of Victoria, Australia. From these groups, 28 secondary schools were initially contacted. Within each region, a random number table was used to determine which schools would be approached. Ultimately, 14 consented to participate.

Following consent from the directors of education and school principals, classroom teachers distributed sealed envelopes through students to their parents. Each envelope contained two sets of documents, one for the mother and one for the adolescent, along with a return envelope. The materials included a letter describing the study, a consent/assent form, and the DBRS and SDQ for both the mother and adolescent. The letters emphasized the importance of completing the questionnaires independently to ensure the reliability of the responses. To reduce potential bias in ratings, the letter specified that the study focused on aspects of each adolescent’s behavior at home, and the questionnaires were not identified by name. The mothers were also asked to indicate their willingness for teachers to complete the DBRS and SDQ for their child. In cases where consent was available, the child’s teacher was subsequently asked to complete these measures. The participants were not compensated in any way for their participation.

In the current study, the participants were required to submit the completed DBRS, and the SDQ ES, CP, and PP scales were graded across the respondents. Approximately 51% of the questionnaires were returned with the required data. Due to ethical constraints, no information was collected from individuals who did not participate.

### 6.4. Statistical Analyses

The SEM approach proposed by Feng and Hancock [51] was employed to examine the incremental validity of the ADHD dimensions in predicting ESs, CPs, and PPs. This analysis adapted the M*plus* syntax provided by Feng and Hancock [51]. To account for gender effects, which are known to influence ADHD symptoms, gender was included as a covariate in all models. The order of predictor entry followed the sequence gender → IM → HY → IA, enabling an assessment of the incremental contribution of each predictor while controlling for the preceding predictors. The robust maximum likelihood ratio (MLR) extraction method was applied for all the incremental validity models.

**Figure 1:**
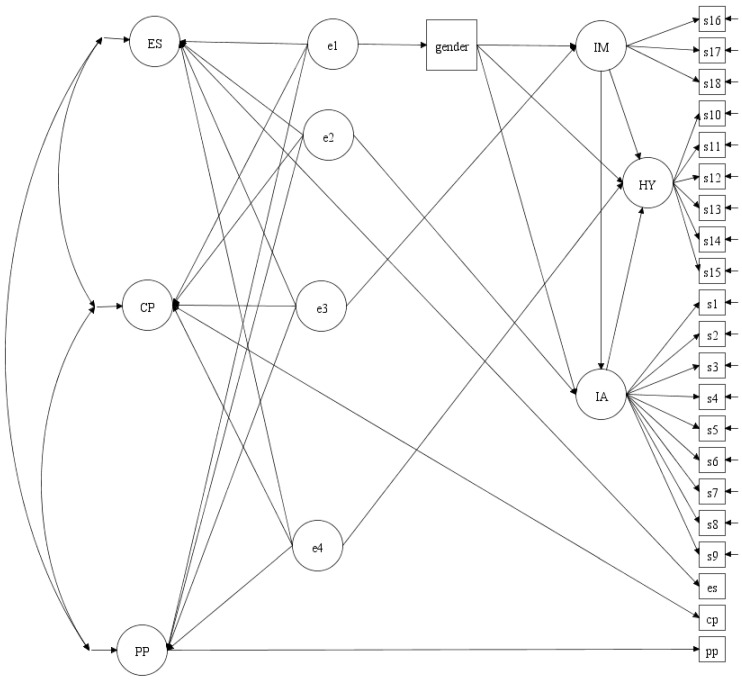
Structural model diagram showing the incremental validity for the prediction of emotional symptoms, conduct problems, and peer problems by (in sequence) gender and ADHD factors of impulsivity, hyperactivity, and inattention. Note: This illustration involves one observed covariate (gender), three latent predictors (IA, HY, and IM), and three latent outcomes (ESs, CPs, and PPs). ES = Emotional Symptom; CP = Conduct Problem; PP = Peer Problem; IA = ADHD inattention symptom group; HY = ADHD hyperactivity symptom group; IM = ADHD impulsivity symptom group; s1 to s18 are the ADHD symptoms in the order presented in DSM-5-TR.

The path analysis model for testing incremental validity is shown in Figure 1. This model included one observed covariate (gender), three latent predictors (IA, HY, and IM), and three latent outcomes (ESs, CPs, and PPs). Each latent predictor, as well as gender, was regressed on the appropriate preceding latent variables. Additionally, phantom variables (e1 to e4) were introduced to capture the residual variance, with each constrained to unit variance. The factor loadings for IA, HY, and IM were constrained to be non-negative, and the latent predictors (IA, HY, and IM) were assigned unit variance, with their loadings estimated freely. In this model, the path coefficients from e1 to e4 to the outcome factors represent semi-partial correlations, showing unique effects of each predictor (gender, IA, HY, and IM) after accounting for the variance contributed to the preceding variables. These coefficients, organized in the Γ matrix, provide statistical evidence for incremental validity. To assess the strength of the predictions, plausible values for ESs, CPs, and PPs were treated as continuous variables (for details on plausible values, see [58]).

The elements in the Γ matrix (semi-partial correlations) were squared to compute the change in the *R*^2^ (Δ*R*^2^) value, with bootstrapping methods to establish 95% confidence intervals (CIs). A significant effect was inferred if the bootstrapping 95% CI did not include zero. For the current study, the confidence intervals for these point estimates were computed using the free software calculator for Δ*R*^2^ [59]. A significant Δ*R*^2^ was interpreted as a significant contribution to incremental validity. A significance Δ*R*^2^ indicates a meaningful incremental validity [35,60]. Since statistical significance depends on the sample size, it does not inherently reflect clinical significance [35,61]. To systematically interpret the Δ*R*^2^ values, Cohen’s [62] criteria were used: 0.26 = substantial, 0.13 = moderate, and 0.02 = weak. Although multiple comparisons were conducted, no adjustments were made, following the example provided by Feng and Hancock [51].

**Figure 2:**
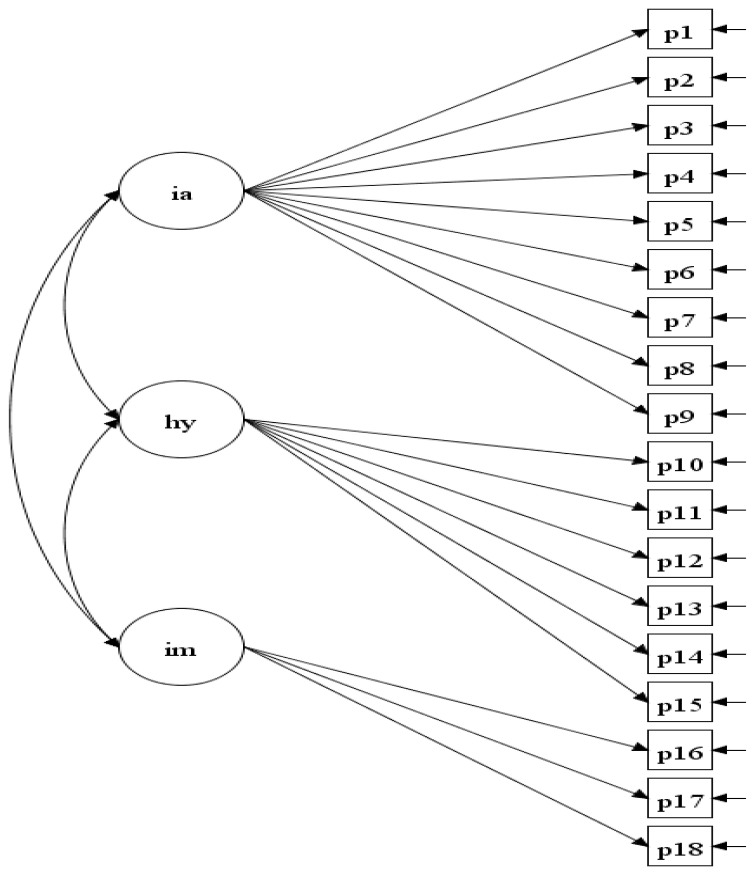
Structural model diagram for the three-factor ADHD model. *Note*: s1 to s18 are the ADHD symptoms in the order presented in DSM-5-TR; IA = ADHD inattention factor; HY = ADHD hyperactivity factor; IM = ADHD impulsivity factor.

Incremental validity is only meaningful when the factorial and discriminant validity of the measurement model is established [51]. Therefore, CFA was initially performed to evaluate the three-factor ADHD model (depicted in Figure 2) and the discriminant validity of its latent factors. The three-factor model was analyzed using the mean and variance-adjusted weighted least squares extraction (WLSMV) method. The model fit was evaluated using three indices: the root mean squared error of approximation (RMSEA), the comparative fit index (CFI), and the Tucker–Lewis Index (TLI). According to Hu and Bentler [60], the model fit was considered good with RMSEA values < 0.06 and acceptable with values < 0.08. For the CFI and TLI, values ≥ 0.95 indicated good fit, and values ≥ 0.90 indicated acceptable fit. In the current study, models were deemed acceptable if the RMSEA value was <0.08 and either the TLI or CFI value was ≥0.90.

Discriminant validity was assessed following the method proposed by Anderson and Gerbing [61], which involves examining the CIs for correlations between the latent factors. Discriminant validity was supported if the 95% CI did not include 1.0. Although a good fit model is not a prerequisite for the valid interpretation of the analysis, the model fit values are presented. The RMSEA, CFI, and TLI thresholds mentioned earlier for WLSMV extraction were used to interpret the model fit.

## 7. Results

### 7.1. Descriptives and Correlations of Study Variables

Table 1 presents the mean scores, *SD,* and intercorrelations of the study variables. In line with our expectations, IA, HY, IM, and CPs were significantly and positively correlated with each other. IA, HY, and IM also showed significant positive correlations with ESs for parent ratings, but these correlations were not observed in the teacher and self-ratings. Gender was significantly and positively correlated with ESs in both teacher and self-ratings. Although not detailed in the table, the ADHD symptoms (across mother, teacher, and adolescent self-ratings) ranged from 0 to 3 for all 18 symptoms. This indicates minimal evidence of range restriction, suggestions sufficient variability in the ADHD ratings.

### 7.2. Factorial Validity of the Three-Factor ADHD Model

Table 2 presents the fit indices for the three-factor ADHD model across parent, teacher, and self-ratings. The CFI and TLI values for all respondents were >0.90, and the RMSEA values were <0.80. According to the recommendations by Hu and Bentler [60] (i.e., RMSEA value < 0.08 and either the TLI or CFI value was ≥0.90), the results support the factorial validity of the three-factor ADHD model across all three respondent groups. Supplementary Tables S1–S3 shows the factor loadings for this model for parent, teacher, and self-ratings, respectively.

### 7.3. Discriminant Validity of the Latent Factors in the Three-Factor ADHD Model

Table 3 displays the correlations between the latent factors (i.e., HI with IA, IM with IA, and IM with HY) across parent, teacher, and self-ratings. The correlations ranged from 0.624 to 0.900, indicating moderate to high associations. However, none of the CIs for these correlations included 1.0, which provides evidence of discriminant validity for the latent factors in the three-factor model. Thus, despite moderate to high correlations, the results support the distinctiveness of the ADHD dimensions across all respondent groups.

### 7.4. Incremental Validity Analyses

The model fit values for the incremental validity test are as follows: For parent ratings, the values were ML*χ*^2^ = 581.800; *df* = 192; *p* < 0.001; CFI = 0.859; TLI = 0.830; and RMSEA = 0.097 (90% CI = 0.088/0.107). For teacher ratings, the values were ML*χ*^2^ = 896.899; *df* = 192; *p* < 0.001; CFI = 0.830; TLI = 0.796; and RMSEA = 0.131 (90% CI = 0.122/0.140). For self-ratings, the values were ML*χ*^2^ = 416.729; *df* = 192; *p* < 0.001; CFI = 0.842; TLI = 0.810; and RMSEA = 0.074 (90% CI = 0.064/0.084). Based on Hu and Bentler’s [60] guidelines, these results indicate that none of the models achieved adequate fit. However, as noted earlier, acceptable model fit is not required for valid interpretation of the incremental validity results.

The parameter estimates for the Γ matrix in the SEM incremental validity analysis are presented in Table 4. These estimates represent the semi-partial correlations for each predictor, with the corresponding Δ*R*^2^ computed by squaring the semi-partial correlations. The columns in Table 4 correspond to the four predictors (i.e., gender, IA, HY, and IM), while the rows represent the path coefficients for each outcome factor (i.e., ESs, CPs, and PPs). More specifically, the third column presents the path coefficients for the outcome predicted by gender; the fourth column provides the path coefficients for IM, controlling for gender; the fifth column shows the path coefficients for the predictions of the outcome factors by the relevant ADHD dimension (HY) after controlling for both gender and IM; and the sixth column presents the path coefficients for IA, controlling for gender, IM, and HY. The semi-partial correlations reflect the unique contributions of each predictor to the outcome variables, independent of the preceding predictors. Significant incremental validity was determined through bootstrapped 95% confidence intervals. If the CIs for the ΔR² did not include zero, the predictor was considered to provide significant incremental variance.

#### 7.4.1. Incremental Validity Based on Parent Ratings

The path coefficients for the SEM for the incremental validity based on self-ratings are shown in Supplementary Figure S1. As shown in the fourth column in Table 4, after controlling for gender, IM significantly and positively predicted 16% of the variance in ESs (∆*R*^2^ = (0.40)^2^ = 0.16, 95% bootstrap CI [0.07/0.25]), 16% of the variance in conduct problems (∆*R*^2^ = (0.40)^2^ = 0.16, 95% bootstrap CI [0.07/0.25]), and 11% of the variance in peer problems (∆*R*^2^ = (0.33)^2^ = 0.11, 95% bootstrap CI [0.00/0.169). According to Cohen’s [62] guidelines for effect sizes (0.26 = substantial, 0.13 = moderate, and 0.02 = weak), these results indicate moderate effects for ESs and CPs and weak effects for PPs.

The fifth column shows that after controlling for gender and IM, HY contributed 14% of the variance in conduct problems (∆*R*^2^ = (0.38)^2^ = 0.14, 95% bootstrap CI [0.06/0.22]), presenting a moderate effect size. In the sixth column, IA explained 10% of the variance for ESs (∆*R*^2^ = (0.32)^2^ = 0.10, 95% bootstrap CI [0.03/0.17]), which corresponds to a weak effect size. No other predications were statistically significant.

#### 7.4.2. Incremental Validity Based on Teacher Ratings

The path coefficients for the SEM for the incremental validity based on self-ratings are shown in Supplementary Figure S2. In the fourth column of Table 4, after controlling for gender, IM significantly and positively predicted 8% of the variance in ESs (∆*R*^2^ = (0.28)^2^ = 0.08, 95% bootstrap CI [0.01/0.15]), 45% of the variance in CPs (∆*R*^2^ = (0.67)^2^ = 0.45, 95% bootstrap CI [0.35/0.55]), and 4% of the variance in PPs (∆*R*^2^ = (0.29)^2^ = 0.04, 95% bootstrap CI [0.00/0.169). These results indicate weak effects for ESs and PPs and a substantial effect for CPs, based on Cohen’s (1988) criteria. No other predications were significant.

#### 7.4.3. Incremental Validity Based on Self-Ratings

The path coefficients for the SEM for the incremental validity based on self-ratings are shown in Supplementary Figure S3. The fourth column in Table 4 indicates that after controlling for gender, IM significantly and positively predicted 8% of the variance in ESs (∆*R*^2^ = (0.29)^2^ = 0.08, 95% bootstrap CI [0.01/0.15]), 18% of the variance in CPs (∆*R*^2^ = (0.43)^2^ = 0.18, 95% bootstrap CI [0.09/0.27]), and 3% of the variance in peer problems (∆*R*^2^ = (0.18)^2^ = 0.09, 95% bootstrap CI [−0.00/0.11]). These results represent weak effects for ESs and PPs and a moderate effect for CPs [62]. The fifth column shows that after controlling for gender and IM, HY contributed 8% of the variance in CPs (∆*R*^2^ = (0.29)^2^ = 0.08, 95% bootstrap CI [0.01/0.15]), indicating a weak effect size. No other predications were significant.

In summary, the results indicated that IM, when controlling for gender, showed varying levels of incremental validity across different informant ratings. Specifically, parent-rated IM contributed moderate variance to ESs and CPs and weak variance to PPs; teacher-rated IM accounted for weak variance in ESs and PPs but provided substantial variance for CPs; and self-rated IM contributed weak variance to ESs and PPs and moderate variance to CPs [62]. Table 5 provides a comprehensive summary of these findings across informants and predictors, highlighting the relative contributions of IM, HY, and IA to the variance explained in ESs, CPs, and PPs.

Additionally, parent-rated IA, after controlling for gender, IM, and HY, explained a weak level of variance in the prediction of ESs. However, teacher- and self-rated IA did not contribute meaningfully to the prediction of ESs, CPs, and PPs. Similarly, neither teacher- nor self-rated IA made significant contributions to the prediction of CPs and PPs.

## 8. Discussion

The present study first evaluated the three-factor ADHD model for parent, teacher, and self-ratings and assessed the discriminant validity of the factors within the model. Following this, the SEM approach proposed by Feng and Hancock [51] was used to examine whether IM provided incremental variance over HY and IA in predicting ESs, CPs, and PPs. Additionally, the study evaluated whether IA offered incremental variance over HY and IA in the predictions of these outcome variables.

For all three respondents, the initial findings supported the three factors and confirmed the discriminant validity of the three factors. These findings align with existing findings [46,48]. Regarding incremental validity, IM was a significant predictor of ESs across all three respondent groups, with moderate effect sizes for parent ratings and small effect sizes for teacher and self-ratings. Additionally, HY did not contribute significant incremental validity to ESs for parent, teacher, or self-ratings when controlling for IM. However, for parent ratings, IA added small incremental validity to ESs when controlling for both IM and HY, but this was not observed in teacher or self-ratings.

For the prediction of CPs, IM was a significant predictor across parent, teacher, and self-ratings, with substantial effect sizes for parent and teacher ratings and a moderate effect size for self-ratings. Additionally, HY contributed moderate incremental validity to CPs for parent ratings and small incremental validity for self-ratings, when controlling for IM. For the prediction of PPs, significant associations with IM were observed for both parent and teacher ratings, although the effect sizes were small. Self-ratings, however, were not significantly associated with PPs. Across all respondents, HY did not add significant incremental validity to the predictions of PPs when controlling for IM, nor did IA contribute significant incremental validity when controlling for both IM and HY.

Overall, the findings suggest that parent-rated IM plays a moderate role in explaining ESs, while teacher and self-rated IM are less significant in this regard. Across all three respondents (i.e., parents, teachers, and adolescents), HY appears to have minimal importance in predicting ESs. For the prediction of CPs, IM ratings for all three sources are important in predicting CPs, whereas its contribution based on self-ratings is small. In contrast, for all three respondents, IA shows little relevance in explaining CPs. Regarding the prediction of PPs, while IM ratings from parents and teachers are associated with PPs, their contributions, along with those of HY and IA, are relatively minor across all three respondents. This suggests that none of the ADHD dimensions, IM, HY, or IA, are particularly important in explaining PPs.

In several respects, the findings of the current study are comparable to those reported by Gomez et al. [24]. That study explored how the hyperactivity, emotional symptoms, conduct problems, peer problems, and prosocial behavior dimensions in the Strength and Difficulties Questionnaire (SDQ; [29]) were associated with the ADHD symptoms groups. Using a CFA S-1 model [30], with IM as the general factor, the study found that for parent ratings, the general factor (IM) significantly and positively predicted all SDQ factors (emotional symptoms, hyperactivity, conduct problems, peer problems, and prosocial factors). Additionally, the IA-specific factor was a significant predictor of HI, peer problems, and prosocial, while the HY-specific factor did not significantly predict any of the SDQ factors. For teacher ratings, the general factor (IM) also predicted hyperactivity, conduct problems, peer problems, and prosocial factors but not emotional symptoms. The IA-specific factor predicted SDQ hyperactivity and prosocial factors, and the HY-specific factor did not predict any of the SDQ dimensions.

Despite the similarities, the current study extends the findings of Gomez et al. [24] in important ways. While Gomez et al. examined the unique associations between ADHD domains and outcomes within a CFA S-1 model, with IM as the general factor, the current study evaluated these relationships using incremental validity to assess how ADHD domains (measured through total scores) predict outcomes. This approach differs because unique associations show the specific relationship between a predictor and an outcome, whereas incremental validity reflects the additional unique variance a predictor contributes after controlling for other relevant variables [34]. Incremental validity analysis offers insights into the relative importance of each predictor in explaining an outcome, which can enhance understanding of critical relations and guide the development of more targeted treatment [36]. Thus, the findings of this study hold important theoretical and practical implications for both understanding ADHD and developing more focused treatment strategies.

Overall, our findings suggest that the HY symptom group plays a minimal role in predicting ESs, CPs, and PPs. This could imply that HY symptom group may not be a key factor in predicting various psychopathology and behavioral issues commonly associated with ADHD. Consequently, it could be argued that the HY symptom is less relevant for understanding ADHD in general. This interpretation aligns with previous studies involving ADHD bifactor models, which reported that HY symptoms were less strongly associated with their specific factor than with the general ADHD factor, especially when compared to IM and IA [42,63,64].

Additionally, a recent study using bifactor exploratory structural equation modeling (ESEM) in adults found that while the overall model fit was acceptable, the HY-specific factor showed weak convergent and divergent validity. In contrast, the general factors, as well as IA and IM factors, demonstrated stronger convergent and divergent validities [65]. Similarly, Barkley and Murphy [66] reported that HY symptoms are less evident in adults with ADHD. This may indicate that while HY is more relevant during childhood, by adolescence, individuals may have learned to better regulate or inhibit their HY behaviors [67].

Given that the findings show that IM and HY symptoms are associated differently with ESs, CPs, and PPs, it may be more appropriate to separate these symptom groups, as was implemented in the ICD-10, rather than combining them into a single group (HY/IM), as proposed in DSM-5-TR, DSM-5, DSM-IV/DSM-IV TR, and ICD-11. This suggest that while HY symptoms have been traditionally viewed as key to ADHD, this may not be the case, as least for adolescents, as has also been suggested for adults [65,66].

The incremental validity findings from this study suggest that while the IM domain can be a major risk factor for ESs and CPs, the IA domain also presents an additional risk factor for ESs, and the HY domain is an additional risk factor for CPs. These results imply that ADHD IA symptoms (and by extension, inattentive presentation) may be more prone to the development of ESs and, consequently, internalizing disorders. In contrast, ADHD HY symptoms (and by extension, the hyperactive–impulsive presentation type) are more likely to be associated with the development of CP and, by extension, externalizing disorders. Therefore, the combined presentation of ADHD may increase one’s susceptibility to both internalizing and externalizing disorders [68,69]. Clinicians may find this information valuable when assessing and treating children with ADHD.

### 8.1. Treatment Implications

Given that our finding indicated that IM is more critical than HY and IA in the predicting psychopathologies and that trait impulsivity underlies IM, it can be argued that directly targeting impulsivity can be an effective approach for treating ADHD. Regarding pharmaceutical treatments, a study comparing clonidine alone, clonidine combined with methylphenidate, and methylphenidate alone in children with ADHD found that clonidine, with or without methylphenidate, was effective in controlling impulsivity, as well as symptoms of oppositional and conduct disorder symptoms [70]. In relation to non-pharmaceutical treatments, cognitive behavioral therapy (CBT) and dialectic behavior therapy (DBT) have demonstrated efficacy in managing impulsivity [71]. Based on these findings, it can be speculated that a treatment strategy combining clonidine (with or without methylphenidate) alongside behavioral therapy or dialectical behavior therapy may be particularly effective in managing ADHD symptoms.

### 8.2. Limitations

Although this study offers valuable insights into the associations between ADHD and common psychopathological problems, several limitations must be acknowledged when interpreting the findings. First, both ADHD and the examined outcomes can be influenced by factors such as age, culture, and existing psychopathologies [72,73,74]. The present study did not control for these factors, which may have confounded the results, particularly in relation to age, as ADHD symptoms vary across different developmental stages [74]. Indeed, previous research indicates that ADHD symptoms tend to decline with age, with HI symptoms showing a steeper decline [75,76].

Second, as the participants were drawn from the general community rather than random selection, the findings may be further confounded and limited in terms of generalization, especially in their application to individuals with clinical levels of ADHD or psychopathologies. Third, the use of self-report questionnaires raises the possibility of common method variance, which may have affected the accuracy of the ratings. Fourth, it remains uncertain whether the findings would replicate using other measures of ADHD, ESs, CPs, and PPs or through clinical interview.

Fifth, since this study was based on a single study, the replication of the findings in future studies is essential. Sixth, the cross-sectional design limits the ability to draw causal inferences from the results. Finally, following the tutorial example provided by Feng and Hancock [51], the study did not adjust for multiple comparisons, increasing the potential for Type 1 error. Despite these limitations, the study has produced novel findings and provides strong support for further research in this area, with careful consideration of the limitations discussed.

When the findings for incremental validity were considered collectively, they indicated that for all three respondent groups (parent, teacher, and self-ratings), IM accounted for more variance than HY and IA in predicting ESs and CPs. In self-ratings, HY did not contribute incremental variance beyond IM in predicting ESs. Furthermore, in parent ratings, IA added additional variance beyond IM and HY in predicting ESs, while HY contributed incremental variance beyond IM in predicting CPs. However, for all three respondents, none of the ADHD dimensions (IM, HY, and IA) predicted PPs. These findings were novel. Nonetheless, one key implication is that the results supported the separation of HY and IM into distinct symptom groups in future studies. Such a separation would likely enhance understanding of ADHD symptoms, particularly regarding the disorder’s comorbidity with other commonly co-occurring psychopathologies, as demonstrated in this study.

## Figures and Tables

**Table 1:** Mean and standard deviation (*SD*) scores and correlations of study variables for parent, teacher, and self-ratings.

	Mean (*SD*)	1	2	3	4	5	6	7	8
Parent ratings									
Gender (1)	1.52 (0.50)	-	−0.060	−0.101	−0.032	−0.048	0.115	0.035	0.025
Age (2)	13.82 (1.29)		-	−0.003	−0.044	−0.068	0.098	−0.023	0.072
Inattention (3)	6.50 (5.79)			-	0.681 **	0.533 **	0.450 **	0.551 **	0.247 **
Hyperactivity (4)	3.13 (3.73)				-	0.701 **	0.295 **	0.539 **	0.166 *
Impulsivity (5)	1.72 (2.12)					-	0.172 *	0.560 **	0.060
Emotional symptoms (6)	2.08 (2.14)						-	0.492 **	0.485 **
Conduct problems (7)	1.52 (1.81)							-	0.323 **
Peer problems (8)	1.41 (1.55)								-
Teacher ratings									
Gender (1)	1.52 (0.50)	-	−0.060	−0.174 *	−0.108	−0.106	0.160 *	.020	0.030
Age (2)	13.82 (1.30)		-	−0.077	−0.082	−0.059	0.110	−0.098	0.043
Inattention (3)	5.67 (6.39)			-	0.717 **	0.521 **	0.249 **	0.567 **	0.281 **
Hyperactivity (4)	2.31(3.44)				-	0.758 **	0.205 **	0.640 **	0.212 **
Impulsivity (5)	0.88 (1.72)					-	0.084	0.509 **	0.174 *
Emotional symptoms (6)	1.22 (1.84)						-	0.310 **	0.481 **
Conduct problems (7)	0.83 (1.53)							-	0.384 **
Peer problems (8)	1.49 (1.79)								-
Self-ratings									
Gender (1)	1.52 (0.50)	-	−0.060	−0.057	0.083	−0.023	0.234 **	0.057	−0.037
Age (2)	13.82 (1.30)		-	0.050	0.015	−0.052	0.086	0.010	0.031
Inattention (3)	5.44 (4.07)			-	0.639 **	0.441 **	0.334 **	0.547 **	0.282 **
Hyperactivity (4)	4.80 (3.36)				-	0.531 **	0.240 **	0.466 **	0.147 *
Impulsivity (5)	1.77 (1.85)					-	0.071	0.402 **	0.088
Emotional symptoms (6)	2.43 (2.06)						-	0.315 **	0.444 **
Conduct problems (7)	2.02 (1.79)							-	0.241 **
Peer problems (8)	1.45 (1.58)								-

Note: ** *p* < 0.01; * *p* < 0.05.

**Table 2:** Fit values for the 3-factor ADHD model for parent, teacher, and self-ratings.

WLSMV*χ*^2^ (*df*)	CFI	TLI	RMSEA (90% CI)
Parent ratings			
291.651 (132)	0.971	0.967	0.075 (0.064 −0.087)
Teacher ratings			
355.423 (132)	0.985	0.982	0.089 (0.078 −0.100)
Self-ratings			
253.929 (132)	0.940	0.931	0.066 (0.053 −0.078)

Note: WLSMV*χ*^2^ = mean and variance-adjusted weighted least squares; CI = confidence interval; RMSEA = root mean square error of approximation; CFI = comparative fit index; TLI = Tucker–Lewis Index. All WLSMV*χ*^2^ values were significant (*p* < 0.001).

**Table 3:** Intercorrelations of the latent factors (and 95% confidence intervals) in the 3-factor ADHD model for parent, teacher, and self-ratings.

	HY	IM
Parent ratings		
IA	0.790 (0.743/0.841)	0.627 (0.541/0.712)
HY	-	0.809 (0.748/0.871)
Teacher ratings		
IA	0.847 (0.805/0.888)	0.695 (0.620/0.769)
HY	-	0.900 (0.856/0.944)
Self-ratings		
IA	0.839 (0.785/0.894)	0.624 (0.519/0.729)
HY	-	0.7409 (0.654/0.827)

Note: IA = inattention; HY = hyperactivity; IM = impulsivity. All correlations were significant (*p* < 0.001).

**Table 4:** Semi-partial correlation and change in *R*^2^ for the prediction of emotional symptoms, conduct problems, and peer problems by (in sequence) gender and ADHD impulsivity, hyperactivity, and inattention.

			ADHD Symptom Group		
		Gender	IM	HY	IA
Parent ratings					
ES	*β* (SE); *z* *R*^2^ (95% CI)	0.11 (0.106); 1.78 0.01 (−0.02/0.04)	0.40 (0.08); 4.92 *** 0.16 (0.07/0.25)	−0.09 (0.09); 0.93 0.01 (−0.02/0.04)	0.32 (0.09); 3.53 *** 0.10 (.03/.17)
CP	*β* (SE); *z* *R*^2^ (95% CI)	0.04 (0.07); 0.54 0.01 (−0.02/0.04)	0.40 (0.10); 3.88 *** 0.16 (0.07/0.25)	0.38 (0.08); 4.79 *** 0.14 (0.06/0.22)	0.18 (0.08); 2.36 * 0.03 (−0.01/0.07)
PP	*β* (SE); *z* *R*^2^ (95% CI)	0.03 (0.07); 0.39 0.01 (−0.01/0.07)	0.33 (0.10); 3.37 ** 0.11 (0.03/0.19)	−0.16 (0.09); 1.85 0.03 (−0.01/0.07)	0.13 (0.09); 1.42 0.02 (−0.02/.06)
Teacher ratings					
ES	*β* (SE); *z* *R*^2^ (95% CI)	0.15 (0.06); 2.49 * 0.02 (−0.02/0.06)	0.28 (0.09); 3.22 ** 0.08 (0.01/0.15)	−0.06 (0.11); 0.60 0.00	0.15 (0.09); 1.74 0.03 (−0.01/.07)
CP	*β* (SE); *z* *R*^2^ (95% CI)	0.02 (0.07); 0.28 0.00	0.67 (0.07); 9.27 *** 0.45 (0.35/0.55)	0.02 (0.10); 0.19 0.00	0.10 (0.08); 1.26 0.01 (−0.01/0.04)
PP	*β* (SE); *z* *R*^2^ (95% CI)	0.03 (0.07); 0.44 0.00	0.20 (0.09); 2.26 * 0.04 (−0.01/0.09)	0.06 (0.09); 0.70 0.00	0.18 (0.09); 2.16 * 0.03 (−0.02/.06)
Self-ratings					
ES	*β* (SE); *z* *R*^2^ (95% CI)	0.23 (0.07); 3.53 *** 0.05 (−0.01/0.11)	0.29 (0.09); 3.53 ** 0.08 (0.01/0.15)	−0.02 (0.08); 0.21 0.00	0.28 (0.09); 2.92 ** 0.03 (−0.01/.07)
CP	*β* (SE); *z* *R*^2^ (95% CI)	0.06 (0.07); 0.28 0.00	0.43 (0.09); 5.05 *** 0.18 (0.09/0.27)	0.29 (0.08); 3.88 *** 0.08 (0.01/0.15)	0.25 (0.11); 2.22 0.01 (−0.01/0.04)
PP	*β* (SE); *z* *R*^2^ (95% CI)	−0.04 (0.07); 0.52 0.00	0.18 (0.09); 1.99 * 0.03 (−0.01/0.07)	0.05 (0.09); 0.60 0.00 (−0.01/11)	0.25 (0.12); 2.10 * 0.06 (−0.00/.12)

Note: ES = emotional symptom; CP = conduct problem; PP = peer problem; IA = inattention; HY = hyperactivity; IM = impulsivity. *** *p* < 0.001; ** *p* < 0.01; * *p* < 0.05.

**Table 5:** Correlations between ADHD symptom dimensions and emotional symptoms, conduct problems, and peer problems.

	Inattention	Hyperactivity	Impulsivity
Parent ratings			
Emotional symptoms	0.44 ***	0.23	0.27 *
Conduct problems	0.27 **	0.09	0.33 **
Peer problems	0.20	0.29	−0.29 *
Teacher ratings			
Emotional symptoms	0.13	0.49	−0.42
Conduct problems	0.10	0.67 *	−0.21
Peer problems	0.47 *	−0.48	0.30
Self-ratings			
Emotional symptoms	0.41 *	0.11	−0.24
Conduct problems	0.49 **	−0.01	0.16
Peer problems	0.49 *	−0.20	−0.02

Note: *** *p* < 0.001; ** *p* < 0.01; * *p* < 0.05.

## Data Availability

The raw data supporting the conclusions of this article will be made available by the authors on request.

## Supplementary Materials

The following supporting information can be downloaded at: https://www.mdpi.com/article/10.3390/pediatric16040095/s1: Supplementary Table S1: Factor loadings for the Three-Factor ADHD Model for Parent Ratings; Supplementary Table S2: Factor loadings for the Three-Factor ADHD Model for Teacher Ratings; Supplementary Table S3: Factor loadings for the Three-Factor ADHD Model for Self Ratings; Supplementary Figure S1: Path Coefficients for Ratings; Supplementary Figure S2: Path Coefficients for Teacher Ratings; Supplementary Figure S3: Path Coefficients for Self-Ratings.
