# Supplementary material for: Incremental Validity of ADHD Dimensions in the Predictions of Emotional Symptoms, Conduct Problems, and Peer Problems in Adolescents Based on Parent, Teacher, and Self-Ratings
Source: Pediatr Rep. 2024 Dec 10;16(4):1115–33. doi: 10.3390/pediatric16040095 (PMC11677613; doi:10.3390/pediatric16040095)
Supplement: Supplementary file 1 [file pediatrrep-16-00095-s001.zip › pediatrrep-3292353-supplementary.pdf]

RMIT Classification: Trusted

Supplementary Figure S3

Path Coefficients for Self-Ratings the Structural Model Diagram Showing the Incremental Validity for the Predictions of Emotional Symptoms, Conduct Problems and Peer Problems by (in Sequence) Gender, and ADHD Factors of Impulsivity, Hyperactivity, and Inattention

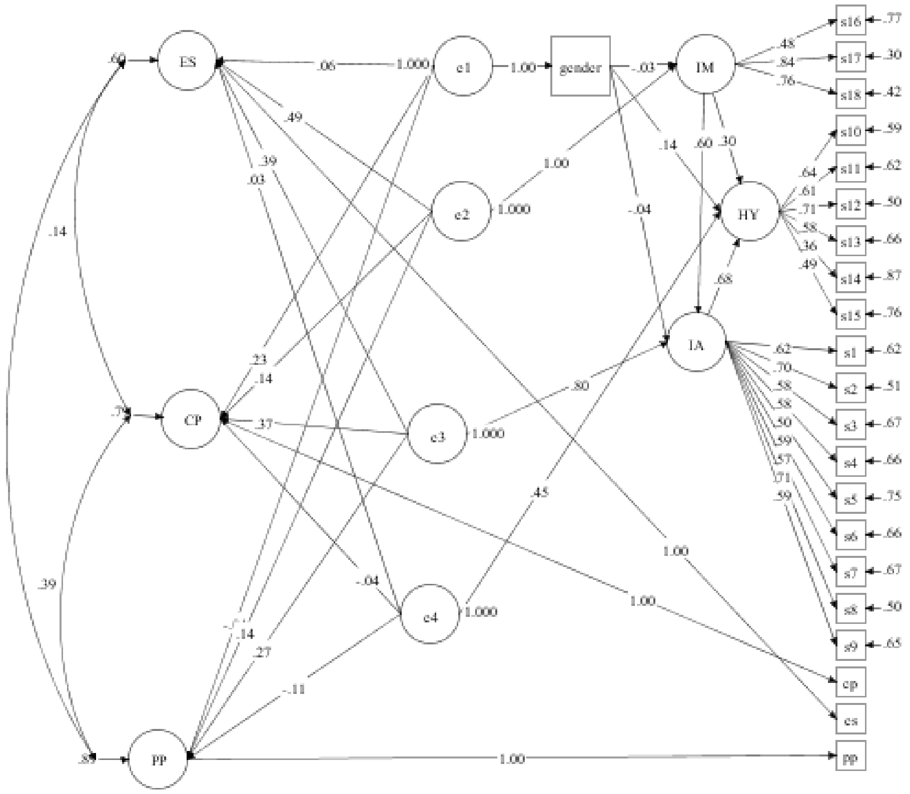

Note. This illustration involves one observed covariate (gender), three latent predictors (IA, HY and IM), and three latent outcomes (ES, CP and PP). ES = Emotional Symptoms; CP = Conduct Problems; PP = Peer Problems; IA = ADHD inattention symptom group; HY = ADHD hyperactivity symptom group; IM = ADHD impulsivity symptom group; s1 to s18 are the ADHD symptoms in the order presented in DSM-5-TR.

RMIT Classification: Trusted

Supplementary Figure S2

Path Coefficients for Teacher Ratings the Structural Model Diagram Showing the Incremental Validity for the Predictions of Emotional Symptoms, Conduct Problems and Peer Problems by (in Sequence) Gender, and ADHD Factors of Impulsivity, Hyperactivity, and Inattention

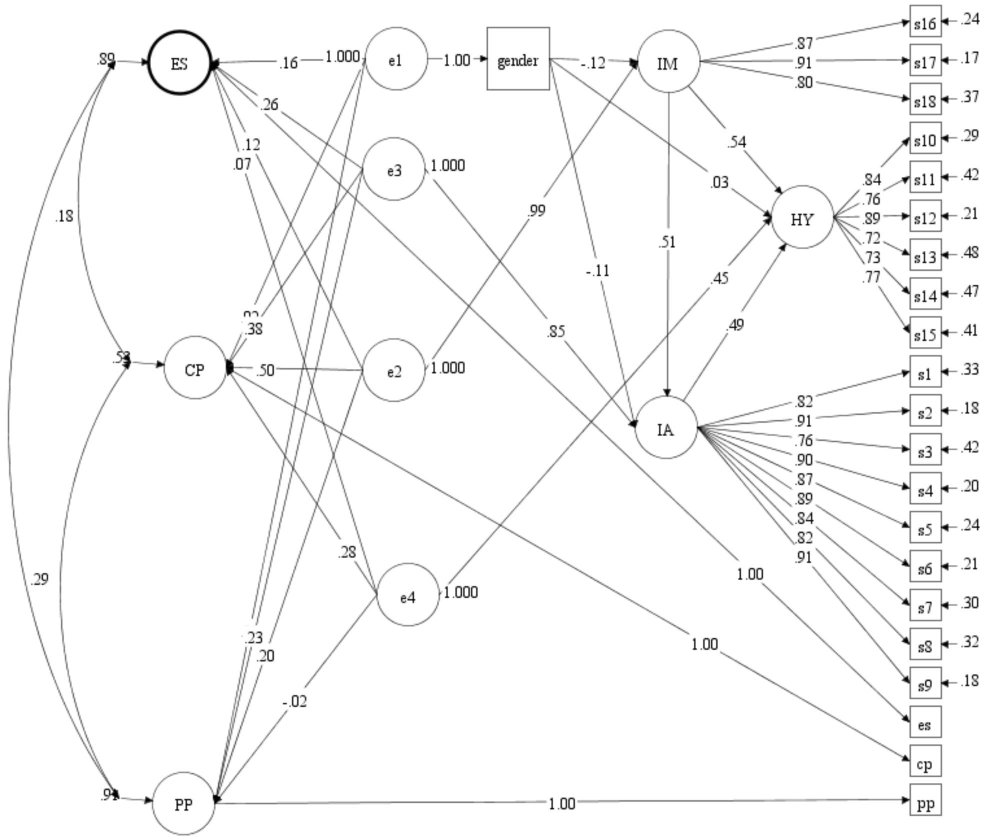

Note. This illustration involves one observed covariate (gender), three latent predictors (IA, HY and IM), and three latent outcomes (ES, CP and PP). ES = Emotional Symptoms; CP = Conduct Problems; PP = Peer Problems; IA = ADHD inattention symptom group; HY = ADHD hyperactivity symptom group; IM = ADHD impulsivity symptom group; s1 to s18 are the ADHD symptoms in the order presented in DSM-5-TR.

RMIT Classification: Trusted

Supplementary Figure S1

Path Coefficients for Ratings the Structural Model Diagram Showing the Incremental Validity for the Predictions of Emotional Symptoms, Conduct Problems and Peer Problems by (in Sequence) Gender, and ADHD Factors of Impulsivity, Hyperactivity, and Inattention

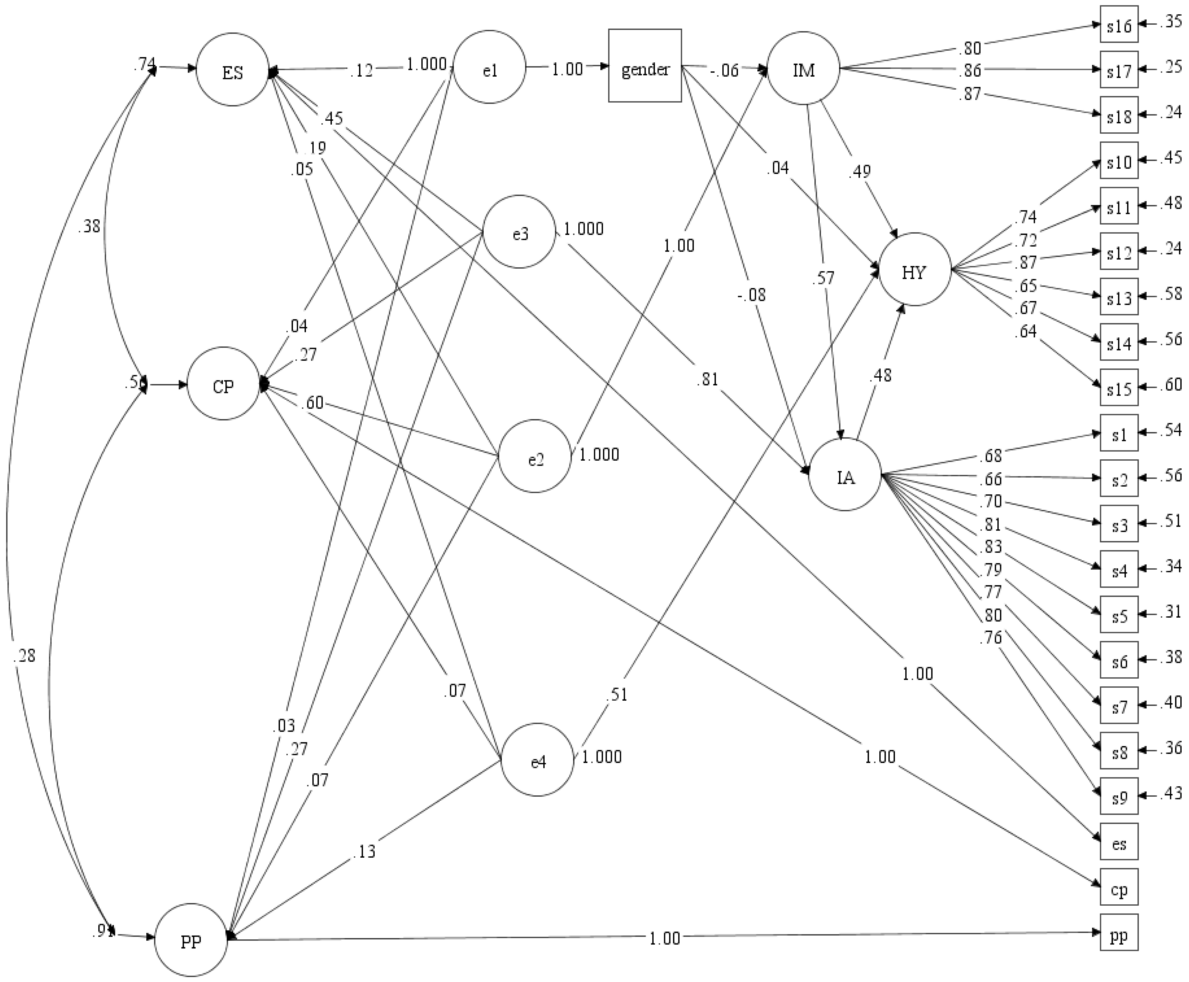

Note. This illustration involves one observed covariate (gender), three latent predictors (IA, HY and IM), and three latent outcomes (ES, CP and PP). ES = Emotional Symptoms; CP = Conduct Problems; PP = Peer Problems; IA = ADHD inattention symptom group; HY = ADHD hyperactivity symptom group; IM = ADHD impulsivity symptom group; s1 to s18 are the ADHD symptoms in the order presented in DSM-5-TR.

RMIT Classification: Trusted

Supplementary Table S1

Factor loadings for the Three-Factor ADHD Model for Parent Ratings

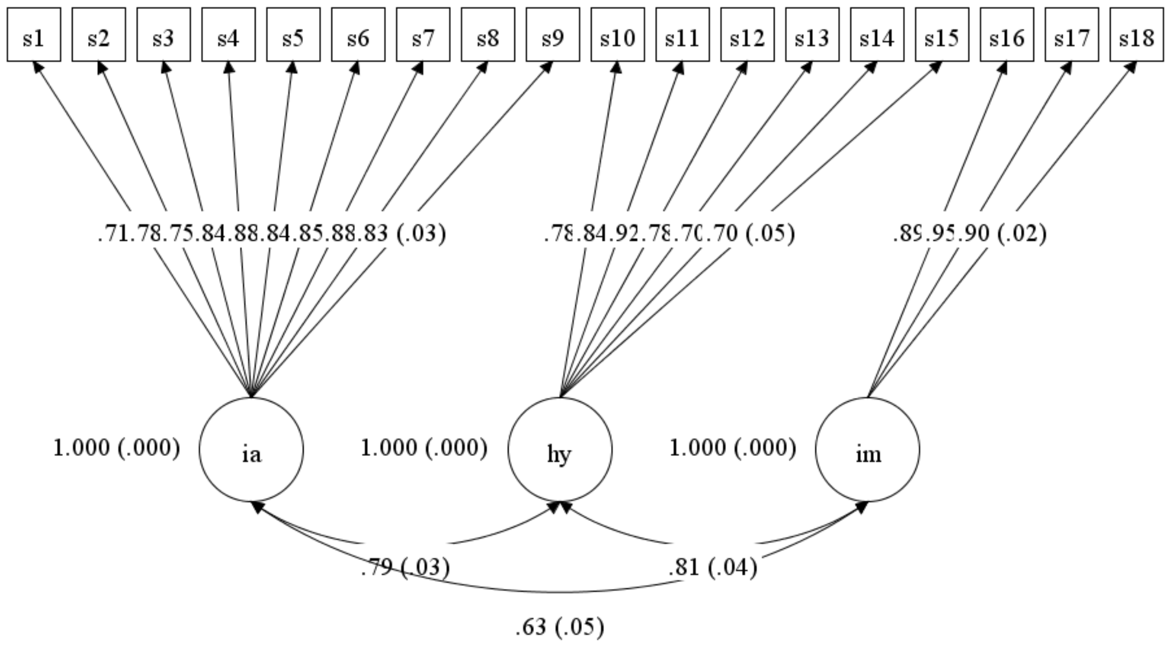

Note. s1 to s18 are the ADHD symptoms in the order presented in DSM-5-TR; IA = ADHD inattention factor; HY = ADHD hyperactivity factor; IM = ADHD impulsivity factor.

RMIT Classification: Trusted

Supplementary Table S2

Factor loadings for the Three-Factor ADHD Model for Teacher Ratings

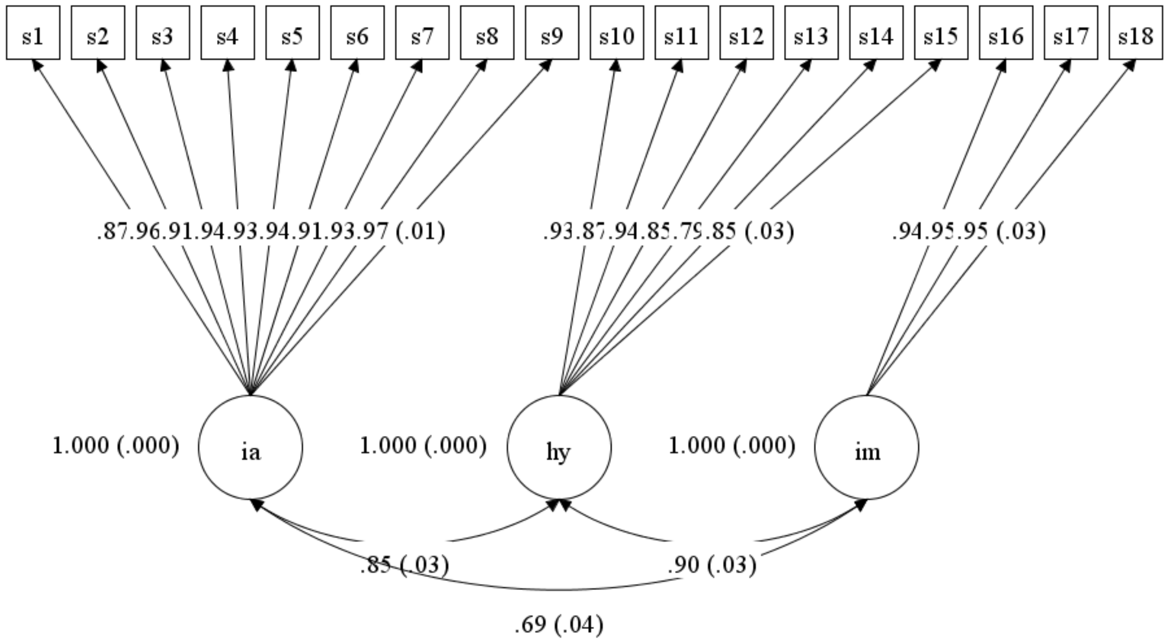

Note. s1 to s18 are the ADHD symptoms in the order presented in DSM-5-TR; IA = ADHD inattention factor; HY = ADHD hyperactivity factor; IM = ADHD impulsivity factor.

RMIT Classification: Trusted

Supplementary Table S3

Factor loadings for the Three-Factor ADHD Model for Self Ratings

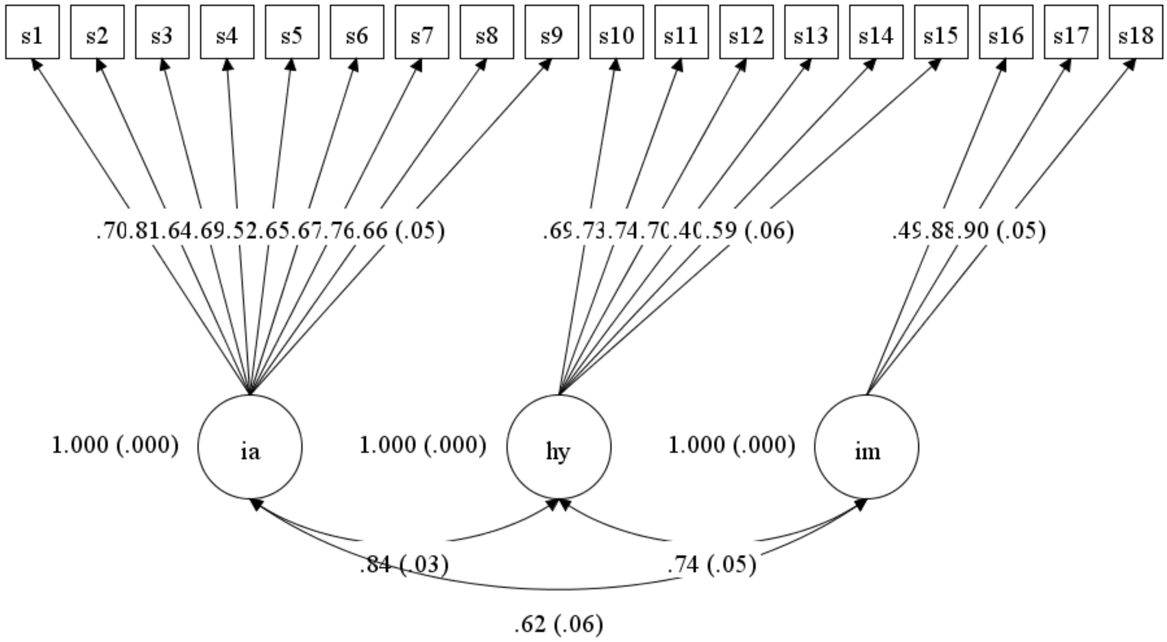

Note. s1 to s18 are the ADHD symptoms in the order presented in DSM-5-TR; IA = ADHD inattention factor; HY = ADHD hyperactivity factor; IM = ADHD impulsivity factor.
